# Epileptic seizures induced by pentylenetetrazole kindling accelerate Alzheimer-like neuropathology in 5×FAD mice

**DOI:** 10.3389/fphar.2024.1500105

**Published:** 2024-10-31

**Authors:** Yulian Zou, Chengyan Wang, Huang Li, Meihua Zhong, Jin Lin, Yan Hu, Zhou Chen, Chen-Ling Gan

**Affiliations:** ^1^ Institute of Immunotherapy, Fujian Medical University, Fuzhou, Fujian, China; ^2^ Institute of Laboratory Animal Center, Fujian Medical University, Fuzhou, China; ^3^ Department of Pharmacy of Fuzhou First General Hospital Affiliated With Fujian Medical University, Fuzhou, China; ^4^ School of Pharmacy, Fujian Medical University, Fuzhou, Fujian, China; ^5^ Public Technology Service Center, Fujian Medical University, Fuzhou, Fujian, China

**Keywords:** seizure, pentylenetetrazole, Alzheimer’s disease, DAPK, ERK, neuropathology, carbamazepine

## Abstract

Clinical studies have shown that epileptic seizures worsen Alzheimer’s disease (AD) pathology and related cognitive deficits; however, the underlying mechanism is unclear. To assess the effects of seizures on the progression of AD, chronic temporal lobe epilepsy was induced in five familial AD mutation (5×FAD) mice by kindling with the chemoconvulsant pentylenetetrazole (PTZ) at 3–3.5 months of age. The amyloidogenic pathway, tauopathy, synaptic damage, neuronal death, neurological inflammatory response and associated kinase signaling pathway dysregulation were examined at 9 months of age. We found that APP, p-APP, BACE1, Aβ and kinase-associated p-tau levels were elevated after PTZ kindling in 5×FAD mice. In addition, PTZ kindling exacerbated hippocampal synaptic damage and neuronal cell death, as determined by scanning electron microscopy and terminal deoxynucleotidyl transferase-mediated deoxyuridine triphosphate nick-end labeling (TUNEL) staining, respectively. Finally, the levels of the neuroinflammation markers GFAP and Iba1, as well as the inflammatory cytokine IL-1β, were increased after PTZ insult. PTZ kindling profoundly exacerbated extracellular regulated kinase (ERK)-death-associated protein kinase (DAPK) signaling pathway overactivation, and acute ERK inhibitor treatment downregulated Aβ production and p-APP and p-tau levels in epileptic 5×FAD mice. In addition, long-term use of the antiseizure drug carbamazepine (CBZ) alleviated seizure-induced accelerated amyloid and tau pathology and ERK-DAPK overactivation in 5×FAD mice. Collectively, these results demonstrate that seizure-induced increases in AD-like neuropathology in 5×FAD mice are partially regulated by the ERK-DAPK pathway, suggesting that the ERK-DAPK axis could be a new therapeutic target for the treatment of AD patients with comorbid seizures.

## Introduction

Alois Alzheimer described the first clinical case of Alzheimer’s disease (AD) in 1907 (Möller and Graeber, 1998). Although the main pathological features of AD are extracellular Aβ deposits and intracellular neurofibrillary tangles of hyperphosphorylated tau protein, recent studies have suggested that neuronal network dysfunction drives AD onset and development (Deng et al., 2022; Maestú et al., 2021; Wang et al., 2023). Seizures, which result from the generation of aberrant neuronal networks (Jean et al., 2023), are common complications in people with AD; the incidence of seizures is 6–10 times greater in AD patients than in healthy older adults, and it is especially high in patients with early-onset AD (Tait et al., 2021; Pandis and Scarmeas, 2012), although generalized convulsive seizures are rare. In addition, AD mice are more susceptible to epilepsy than wild-type mice of the same age (Garcia-Cabrero et al., 2013; Westmark et al., 2008; Bezzina et al., 2015; Chan et al., 2015). Several clinical studies have shown that seizure activity exacerbates cognitive dysfunction in AD patients (Vossel et al., 2016; Vossel et al., 2013); however, the underlying cellular and molecular mechanisms are still unclear.

Temporal lobe epilepsy (TLE), a devastating seizure disorder, and AD are two different neurological diseases. However, a growing body of evidence suggests that they share several pathologic features, including temporal lobe atrophy, neuronal death, and neuroinflammation (Duan et al., 2020; Kaestner et al., 2021). Emerging evidence from both clinical studies and animal experiments suggests that epileptic seizures trigger classic AD-like pathology. Patients with drug-resistant epilepsy exhibit imaging features of profound brain aging, including a significant increase in Aβ42 levels, similar to the pathological features of AD (Pardoe et al., 2017; Dabbs et al., 2012). In addition to amyloid pathology, the hyperphosphorylation and aggregation of tau have also been reported in the brains of epilepsy patients and animal models of epilepsy (Gourmaud et al., 2020; Crespo-Biel et al., 2007; Liang et al., 2009; Tian et al., 2010; Jones et al., 2012; Liu et al., 2016). These associations suggest that common signaling pathways may be involved in these two types of brain dysfunction. One potential link between TLE and AD is extracellular regulated kinase (ERK)-death-associated protein kinase (DAPK) axis, which has been reported to be overactivated in patients with TLE and AD (Gan et al., 2022; Gan et al., 2021; You et al., 2017; Chun et al., 2022; Ephrame et al., 2023). DAPK, an important serine/threonine protein kinase, regulates Aβ generation, tauopathy, and neuronal cell death in AD (You et al., 2017; Kim et al., 2016; Zhang et al., 2022; Kim et al., 2014; Wang et al., 2022b; Wang et al., 2022a; Zhang, Kim, and Lee, 2024). Our recent study revealed that DAPK drives epileptic seizures induced by pentylenetetrazole (PTZ) (Gan et al., 2021) and that blockade of the ERK‒DAPK signaling pathway inhibits kainic acid (KA)-induced epileptogenesis (Gan et al., 2022). Considering that DAPK is a downstream signaling molecule of ERK (Chen et al., 2005) and that both DAPK and ERK play similar roles in AD and epilepsy (Chun et al., 2022; Nateri et al., 2007), we hypothesized that seizure-induced exacerbation of AD may involve dysregulation of the ERK‒DAPK pathway. Therefore, in the present study, we used a 5×FAD mouse model to explore the potential impact of experimentally induced chronic TLE on the progression of AD neuropathology and the ERK‒DAPK signaling pathway. PTZ, a central nervous system stimulant, is mainly used to establish a chronic kindling model of epilepsy. We found that kindled seizures induced by PTZ aggravated amyloid and tau pathology, synaptic and neuronal damage, inflammatory responses and ERK‒DAPK axis overactivation in 5×FAD mice and that acute ERK inhibitor treatment alleviated the aggravated amyloid and tau pathology induced by PTZ kindling. Moreover, the antiseizure drug CBZ alleviated seizure-induced accelerated amyloid and tau pathology and ERK-DAPK overactivation in 5×FAD mice. Taken together, these findings provide new insights into the mechanisms underlying the interaction between AD and epilepsy.

## Materials and methods

### Materials

PTZ was purchased from MilliporeSigma (St. Louis, MO, United States), and the ERK inhibitor SL327 (HY-15437) and CBZ (HY-B0246) were obtained from MedChemExpress (NJ, United States).

### Animals

Male wild-type (WT) and 5×FAD mice on the C57BL/6 background were used for all the animal experiments. The method for generating 5×FAD mice was previously reported (Zou et al., 2021).

### PTZ dosing paradigms

To study the effects of chronic hyperexcitability on AD progression, we generated a model of chronic kindling epilepsy by injecting PTZ into 5×FAD mice as described in a previous study (Gourmaud et al., 2022). PTZ was dissolved in 0.9% saline for use. For the kindling paradigm, 5×FAD mice were injected intraperitoneally (i.p.) with a subconvulsive dose of PTZ (35 mg/kg) every other day for 2 weeks, and seizure severity after each PTZ injection was scored as follows (Gan et al., 2021): stage 0: normal behavior, no abnormality; stage 1: immobilization; stage 2: head nodding, partial myoclonus; stage 3: continuous forelimb myoclonus, myoclonic jerks; stage 4: rearing, chronic seizure; stage 5: generalized tonic‒clonic seizures and jumping; and stage 6: death. The mice were considered fully kindled when they exhibited convulsive seizures (at least stage 4) on three consecutive days.

### Immunohistochemical analysis

Deeply anesthetized mice were perfused with 0.1 M PBS (pH = 7.4). Their brains were removed and postfixed in 4% paraformaldehyde for 24 h. Paraffin-embedded sections were dewaxed with xylene and rehydrated with graded alcohol solutions. Antigen retrieval was performed with citric acid buffer at 120°C for 10 min. Then, the sections were blocked with 3% H_2_O_2_ and 10% fetal bovine serum to block non-specific reaction. The sections were subsequently incubated with primary antibody overnight at 4°C, followed by incubation with an HRP-conjugated secondary antibody for 1 h at room temperature. Finally, the sections were stained with DAB for 2–5 min and hematoxylin for 5 min. Staining was detected via an optical microscope and quantified via ImageJ software. IHC results for Aβ, p-tau, p-DAPK and DAPK were quantified via the H score results (Liu et al., 2023). H-score = ∑ (pi×i), where pi represents the positive signal area/total area and i represents the staining intensity score. IHC results for GFAP and Iba1 were quantified as previously described (Bashir et al., 2023). The primary antibodies used in the immunohistochemical analysis included anti-β-amyloid (BioLegend; SIG-39320; 1:100), anti-p-tau231 (Abcam; ab151559; 1:200), anti-p-tau262 (Thermo Fisher Scientific; 44-750G; 1:100), anti-p-tau202/205 (Servicebio; GB113883-100; 1:800), anti-GFAP (Proteintech Group; 16825-1-AP; 1:400), anti-Iba1 (Proteintech Group; 10904-1-AP; 1:100), anti-DAPK (Proteintech Group; 25136-1-AP; 1:200) and anti-p-DAPK735 (Invitrogen; PA5-105872; 1:100) antibodies.

### Western blotting

Mouse tissues were lysed with RIPA buffer to obtain tissue homogenates. The boiled samples were loaded onto SDS‒PAGE gels and then transferred to polyvinylidene fluoride (PVDF) membranes. The membranes were blocked with 5% BSA for 30 min at room temperature and then probed with primary antibody overnight at 4°C, followed by incubation with an HRP-conjugated secondary antibody for 1 h at room temperature. Finally, the blots were scanned with a ChemiDoc XRS + system and analyzed via ImageJ software. The primary antibodies used for Western blotting included anti-DAPK (Millipore Sigma; D2178; 1:1,000), anti-p-DAPK735 (Invitrogen; PA5-105872; 1:1,000), anti-ERK (Cell Signaling Technology; 4695; 1:10,000), anti-p-ERK202/204 (Cell Signaling Technology; 9101; 1:10,000), anti-MLC (Abcam; ab233152; 1:1,000), anti-p-MLC19 (Cell Signaling Technology; 3675; 1:1,000), anti-p-tau231 (Abcam; ab151559; 1:1,000), anti-p-tau262 (Thermo Fisher Scientific; 44-750G; 1:500), anti-p-tau202/205 (Servicebio; GB113883-100; 1:2000), anti-tau (Proteintech Group; 10274-1-AP; 1:1,000), anti-PSD95 (Proteintech Group; 20665-1-AP; 1:1,000), anti-Synapsin I (Proteintech Group; 20258-1-AP; 1:1,000), anti-GFAP (Proteintech Group; 16825-1-AP; 1:1,000), anti-Iba1 (Proteintech Group; 10904-1-AP; 1:1,000), anti-APP (Proteintech Group; 25524-1-AP; 1:1,000), anti-p-APP668 (Cell Signaling Technology; 3823S; 1:1,000), anti-BACE1 (Proteintech Group; 12807-1-AP; 1:1,000), anti-Cleaved Caspase 3 (Cell Signaling Technology; 9661; 1:1,000), anti-IL-1β (Abcam; ab254360; 1:1,000) and anti-β-actin (MilliporeSigma; A5441; 1:50,000) antibodies.

### Transmission electron microscopy

Harvested fresh hippocampal tissue was immediately perfused with 2.5% glutaraldehyde in 0.2 M PBS (pH = 7.0–7.5). Tissue slabs of 1 mm^3^ were sampled from the hippocampus via an anatomical microscope and fixed in 2.5% glutaraldehyde for 2 h at 4°C. After rinsing in PBS (0.1 M, pH 7.4) 3 times, the samples were fixed in 0.1 M osmium tetroxide at 4°C for 7 h. After dehydration in ascending ethanol and acetone solutions, the blocks were embedded in epoxy resin and cut into sections (60–80 nm), and the sections were stained with 2% uranyl acetate and then 2.6% lead citrate. The sections were dried overnight at room temperature and then photographed via transmission electron microscopy, and the thickness of the postsynaptic density (PSD) was measured via ImageJ software.

### Hematoxylin‒eosin (HE) staining and terminal deoxynucleotidyl transferase dUTP nick-end labeling (TUNEL)

For HE staining, paraffin-embedded sections were dewaxed with xylene and rehydrated with graded alcohol solutions. Then, the sections were stained with eosin and hematoxylin. Histopathological damage was detected via optical microscopy. For TUNEL staining, a TUNEL assay kit (Beyotime Biotechnology, Shanghai, China) was used to assess apoptosis in brain tissues (Zhao et al., 2020). In brief, paraffin-embedded sections were deparaffinized with xylene, rehydrated with graded alcohol solutions, and then treated with proteinase K for 20 min and 3% H_2_O_2_ for 10 min at room temperature. The sections were subsequently incubated with a biotin-dUTP/TdTase mixture for 1 h at 37°C followed by incubation with streptavidin-HRP for 30 min at room temperature, after which the sections were stained with DAB for 3 min and hematoxylin for 5 min. Staining was quantified via ImageJ software.

### Cell culture and apoptosis analysis

The SHSY5Y and SHSY5Y-APP human neuroblastoma cell lines were cultured in DMEM/F12 medium (1:1 ratio) supplemented with 10% fetal bovine serum, 100 U/ml penicillin, and 100 μg/mL streptomycin. The cells were cultured in a humidified incubator with 5% CO2 at 37°C. SHSY5Y cells were collected from a 48-well plate and washed with phosphate-buffered saline in tubes. The cells were then incubated with Annexin V-FITC and PI-PerCP-Cy5.5 at 4°C for 15 min following the manufacturer’s protocol. The apoptosis of SH-SY5Y cells was assessed via flow cytometry and analyzed with FlowJo X software.

### Soluble Aβ42 expression

The amount of soluble Aβ was assessed with an Aβ42 ELISA Kit (FUJIFILM Wako Pure Chemical Corporation, Osaka, Japan) as previously described (Wang et al., 2022b).

### Statistical analysis

Statistical analysis was performed via GraphPad Prism 8.0 software. The data are presented as the means ± SDs. The data were analyzed via a standard two-tailed Student’s t-test for two-group comparisons and one-way ANOVA with Dunnett’s *post hoc* test.

## Results

### PTZ kindling worsens amyloid pathology in 5×FAD mice

**FIGURE 1:**
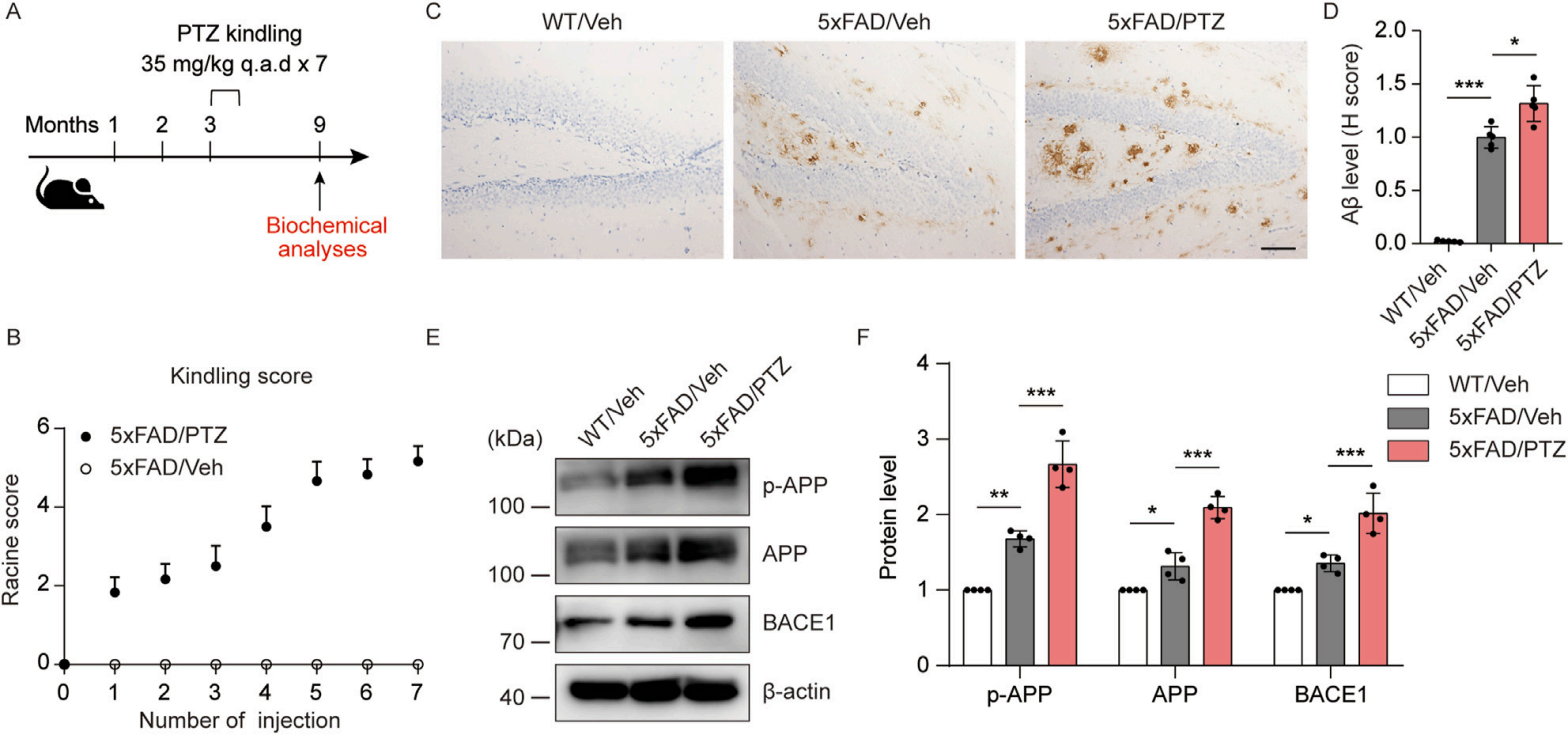
PTZ kindling worsens amyloid pathology in 5×FAD mice. **(A)** Experimental design: 5×FAD mice (C57BL/6, P90‒P110, n = 12‒18/group) were treated with a subconvulsive dose of PTZ (35 mg/kg) every other day (7 injections for 2 weeks), and brain tissue was harvested for analysis 6 months after the last PTZ treatment. **(B)** The mean seizure grades of 5×FAD mice treated with vehicle or PTZ. **(C)** Immunohistochemical staining with an anti-Aβ antibody was conducted on paraffin-embedded hippocampal sections from WT/Veh, 5×FAD/Veh and 5×FAD/PTZ mice. Scale bar, 100 μm. **(D)** Quantification of Aβ42 staining intensity. N = 5. **(E, F)** Hippocampal lysates were subjected to Western blot analysis with anti-APP, anti-p-APP, anti-BACE1 or anti-β-actin antibodies. N = 4. Statistical significance was determined by one-way ANOVA with Dunnett’s multiple comparisons test (*p < 0.05; **p < 0.01; ***p < 0.001). The data are presented as the means ± SD.

To determine how kindled seizures exacerbate pathological changes in AD, 5×FAD mice were kindled with PTZ when they were asymptomatic (3–3.5 months of age) (Gourmaud et al., 2022), and the effects of kindled seizures on AD neuropathology were assessed 6 months later (Figure 1A). During PTZ-induced kindling, 5×FAD mice presented a progressive increase in seizure grade, beginning with normal behaviors (seizure score = 0) and eventually progressing to the first typical convulsive seizure (seizure score ≥4; Figure 1B). The mice were considered fully kindled when they exhibited convulsive seizures on three consecutive days. The focus of this study was to analyze changes in indicators in the hippocampal region after PTZ kindling, as lesions in this brain region significantly affect cognition and behavior in AD patients. We first analyzed the effects of PTZ stimulation on amyloid pathology in the brains of the mice. As shown by the immunohistochemical results, almost no Aβ deposits were detected in the brain tissue of WT mice, whereas scattered Aβ deposits were detected in 5×FAD mice regardless of whether they were subjected to kindling. Consistent with the increase in seizure severity, PTZ kindling led to an increased amyloid plaque burden in the hippocampus of 5×FAD mice (Figures 1C, D). Aβ is a metabolite of APP. To determine whether the increase in Aβ42 expression was associated with a change in APP expression, hippocampal APP levels were quantified via Western blotting. APP expression in the hippocampus was elevated in naïve 5×FAD mice compared with WT mice, and kindled seizures further increased APP expression in the 5×FAD group (Figures 1E, F). In addition, along with an increase in hippocampal APP expression, we detected a corresponding increase in APP phosphorylation at Thr668 (Figures 1E, F), a posttranslational modification that favors the metabolism of APP, after PTZ injection. BACE1 is one of the most important enzymes involved in APP metabolism. Western blot analysis revealed that BACE1 expression was also elevated in 5×FAD mice compared with WT mice, and PTZ insult further elevated the expression of BACE1 in the 5×FAD group (Figures 1E, F). In addition, chronic PTZ insult did not affect p-APP, APP, or BCAE1 levels in WT mice (Supplementary Figures S1A, B), suggesting that PTZ has no long-term effect on the amyloid pathway in WT mice. SHSY5Y-APP neuroblastoma cells have been widely used as an *in vitro* model to mimic AD pathology, and we next investigated whether the amyloid pathway can be aggravated by PTZ in cultured cells. As shown in Supplementary Figures S2A–C, p-APP, APP and BACE1 levels and Aβ42 generation were increased after PTZ treatment in SHSH5Y-APP cells. Taken together, these results suggest that PTZ kindling leads to increased amyloid pathology in 5×FAD mice, possibly via increased APP production and metabolism.

### PTZ kindling exacerbates tau pathology in 5×FAD mice

**FIGURE 2:**
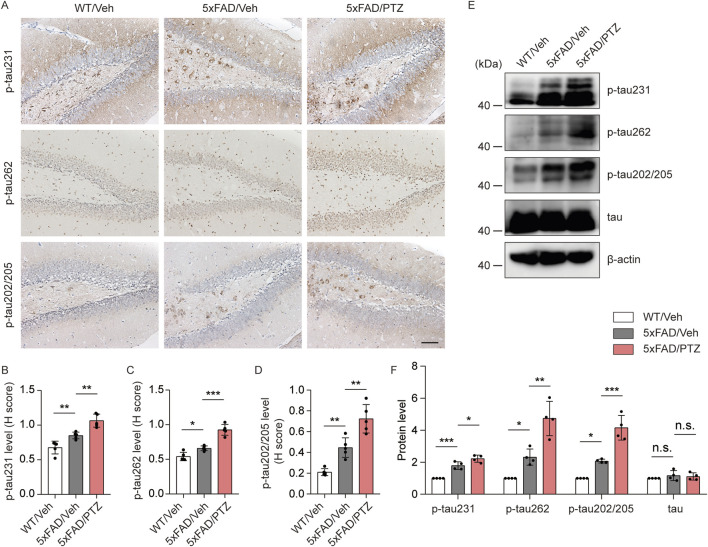
PTZ kindling exacerbates tau pathology in 5×FAD mice. **(A)** Immunohistochemical staining with anti-p-tau231, anti-p-tau262 or anti-p-tau202/205 antibodies were conducted on paraffin-embedded hippocampal sections from WT/Veh, 5×FAD/Veh and 5×FAD/PTZ mice. Scale bar, 100 μm. **(B–D)** Quantification of p-tau231, p-tau262 or p-tau202/205 staining intensity. N = 5. **(E, F)** Hippocampal lysates were subjected to Western blot analysis with anti-p-tau231, anti-p-tau262, anti-p-tau202/205, anti-tau, or anti-β-actin antibodies. N = 4. Statistical significance was determined by one-way ANOVA with Dunnett’s multiple comparisons test (*p < 0.05; **p < 0.01; ***p < 0.001). n.s., not significant. The data represent the mean ± SD.

Hyperphosphorylation of tau is another histopathological feature of AD. Although the 5×FAD mouse strain is the classic Aβ-related AD model, several recent studies have shown that the accumulation of Aβ leads to tau phosphorylation and *vice versa*. Moreover, we and others have shown that elevated levels of tau phosphorylation can be detected in 5×FAD mice, although NFTs cannot be observed (Zou et al., 2021). To examine whether seizures exacerbate tauopathy, both PTZ-treated and vehicle-treated 5×FAD mice were sacrificed at the indicated time points, and brain tissues were collected for further biochemical analyses. As shown in Figures 2A–D, immunohistochemistry data revealed significant hyperphosphorylation of tau protein at multiple sites, including mitogen-activated protein kinase (MAPK)-regulated phosphorylation sites (Ser202/Thr205), DAPK-regulated phosphorylation sites (Ser262) and glycogen synthase kinase 3-regulated phosphorylation sites (Thr231), in 5×FAD mice compared with WT mice, and PTZ kindling further exacerbated tau hyperphosphorylation at these sites in the 5×FAD group, whereas chronic PTZ treatment did not affect p-tau levels in WT mice, as shown in Supplementary Figures S1A, B. In addition, the WB data revealed that the levels of site-specific p-tau were increased in the hippocampus of 5×FAD mice after PTZ simulation (Figures 2E, F). In conclusion, PTZ kindling aggravates tau pathology in 5×FAD mice, which may be related to the overactivation of multiple kinases.

### PTZ kindling exacerbates neuronal damage in 5×FAD mice

**FIGURE 3:**
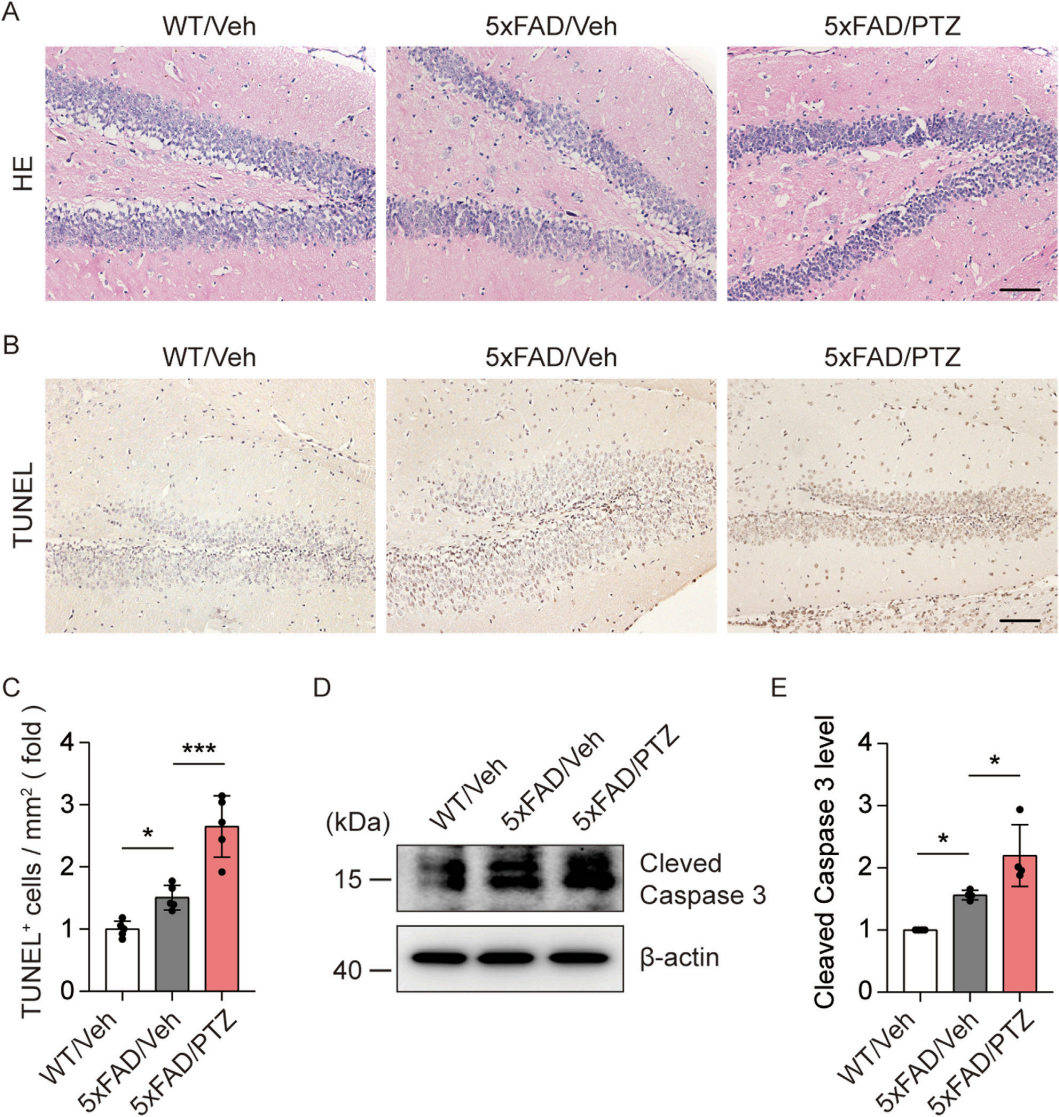
PTZ kindling exacerbates neuronal death in 5×FAD mice. **(A, B)** Representative images of HE staining and TUNEL staining from WT/Veh, 5×FAD/Veh and 5×FAD/PTZ mice. Scale bar, 100 μm. **(C)** Quantification of the TUNEL staining intensity. N = 5. **(D, E)** Hippocampal lysates were subjected to Western blot analysis with anti-Cleaved Caspase 3 or anti-β-actin antibodies. N = 4. Statistical significance was determined by one-way ANOVA with Dunnett’s multiple comparisons test (*p < 0.05; ***p < 0.001). The data represent the mean ± SD.

Aβ accumulation leads to neuronal damage and the progressive loss of neurons in the brain (Gourmaud et al., 2022). We examined histological changes via HE staining. As shown in Figure 3A, nuclear atrophy and neuronal atrophy were observed in 5×FAD mice regardless of whether they were subjected to PTZ kindling. To examine whether kindled seizures exacerbate neuronal damage, we examined the number of apoptotic cells in hippocampal tissues via TUNEL staining. There were significantly more TUNEL-positive neurons in the hippocampi of 5×FAD mice than in those of WT mice, and PTZ insult significantly aggravated neuronal apoptosis in the 5×FAD group (Figures 3B,C), whereas chronic PTZ administration did not induce neuronal damage in WT mice (Supplementary Figures S1C, D). In addition, WB analysis revealed that PTZ kindling upregulated the expression of the apoptosis-related marker Cleaved Caspase 3 (Figures 3D, E). To investigate whether PTZ can aggravate Aβ-induced cell death in neuronal cultures, another AD model was established in SHSY5Y cells via treatment with Aβ. As shown in Supplementary Figures S2D, E, the apoptotic rate was 1.99% ± 0.45% in the control group, whereas after exposure to 15 μM Aβ for 24 h, the apoptotic rate of the SHSY5Y cells increased to 12.53% ± 0.90%, and PTZ insult aggravated Aβ-induced cell death. These results clearly show that PTZ kindling exacerbates neuronal cell death in 5×FAD mice.

### PTZ kindling worsens synaptic damage in 5×FAD mice

**FIGURE 4:**
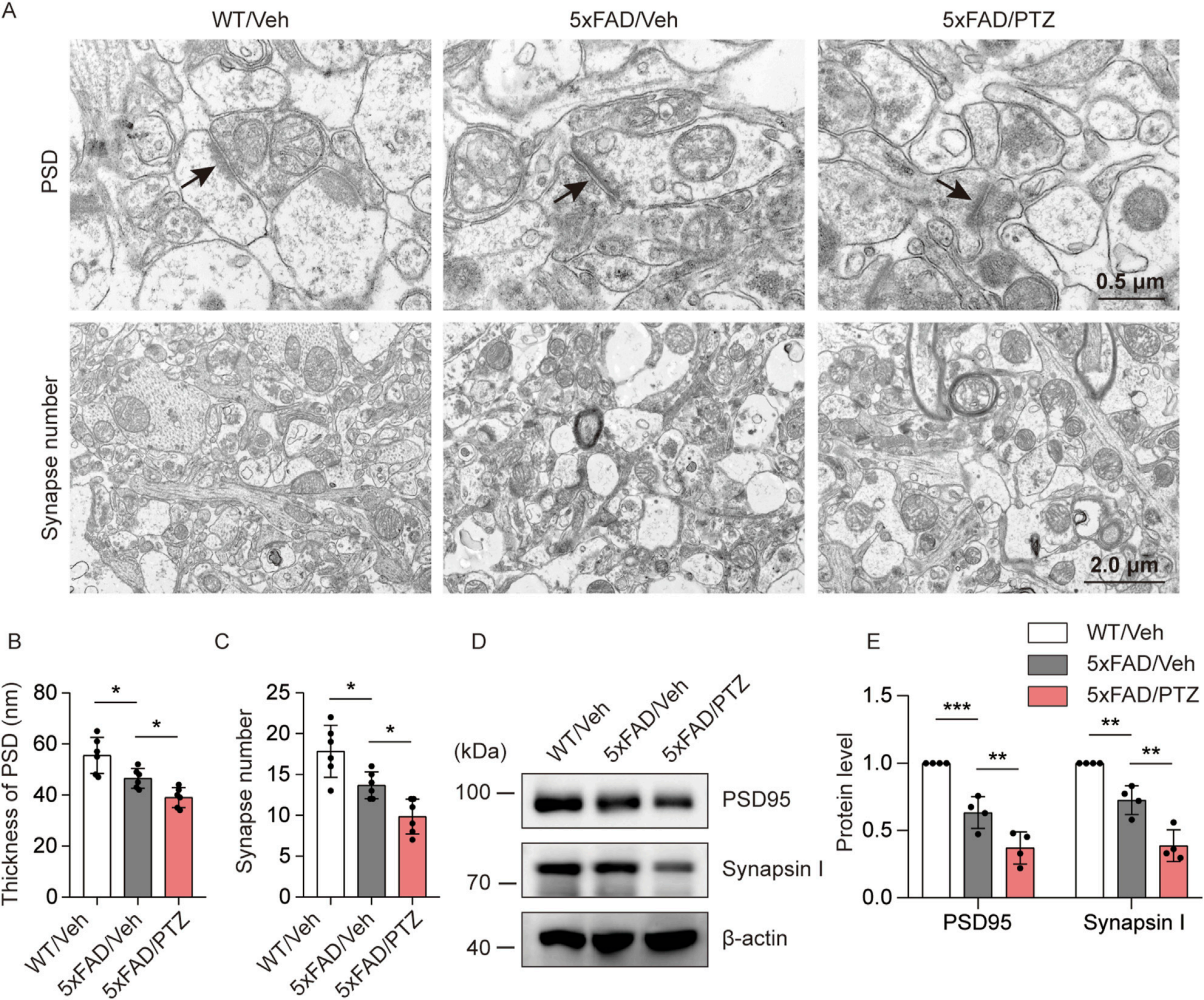
PTZ kindling worsens synaptic damage in 5×FAD mice. **(A)** Representative transmission electron microscopy images of WT/Veh, 5×FAD/Veh and 5×FAD/PTZ mice. **(B, C)** Quantification of PSD thickness and synapse number. N = 6. **(D, E)** Hippocampal lysates were subjected to Western blot analysis with anti-PSD95, anti-Synapsin I or anti-β-actin antibodies. N = 4. Statistical significance was determined by one-way ANOVA with Dunnett’s multiple comparisons test (*p < 0.05; **p < 0.01; ***p < 0.001). The data represent the mean ± SD.

To investigate the effects of seizure activity on synaptic damage, the hippocampal synaptic ultrastructure was visualized via transmission electron microscopy. PSD thickness in the hippocampal region was significantly lower in the 5×FAD group than in the normal WT group, and PTZ treatment further decreased PSD thickness (Figures 4A, B). Moreover, naïve 5×FAD mice had significantly fewer synapses than WT mice did, and PTZ treatment further exacerbated synaptic loss within the 5×FAD group (Figures 4A, C). PSD95 and Synapsin I are synapse marker proteins. WB analysis also revealed that kindling seizures downregulated PSD95 and Synapsin I expression (Figures 4D, E), whereas repeated PTZ administration did not affect PSD95 or Synapsin I expression in WT mice (Supplementary Figures S1A, B), suggesting that PTZ kindling exacerbates synaptic damage in 5×FAD mice.

### PTZ kindling worsens glial reactivation in 5×FAD mice

**FIGURE 5:**
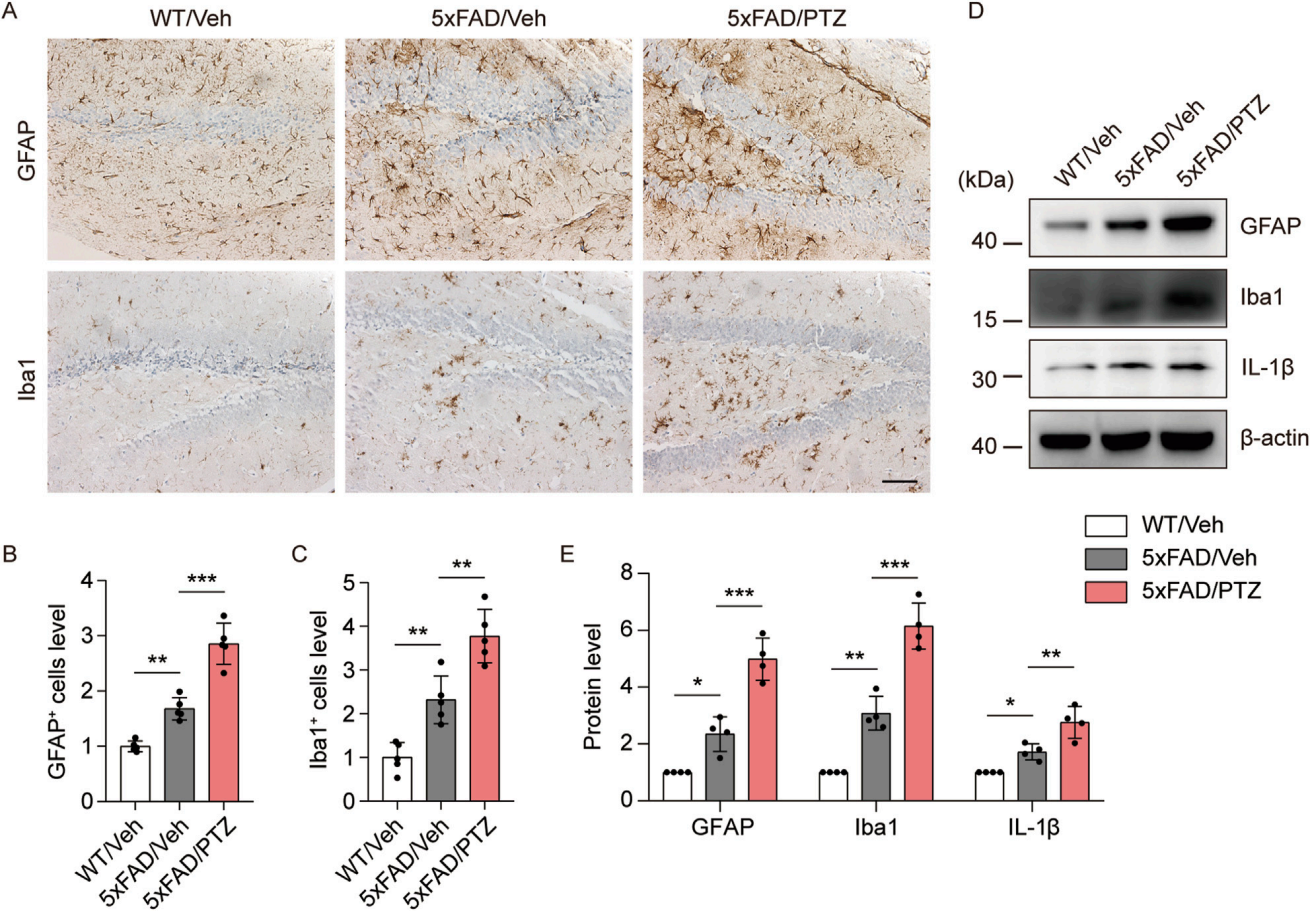
PTZ kindling exacerbates the neuroinflammatory response in 5×FAD mice. **(A)** Immunohistochemical staining with anti-GFAP or anti-Iba1 antibodies was conducted on paraffin-embedded hippocampal sections from WT/Veh, 5×FAD/Veh and 5×FAD/PTZ mice. Scale bar, 100 µm. **(B, C)** Quantification of anti-GFAP or anti-Iba1 staining intensity. N = 5. **(D, E)** Hippocampal lysates were subjected to Western blot analysis with anti-GFAP, anti-Iba1, anti-IL-1β or anti-β-actin antibodies. N = 4. Statistical significance was determined by one-way ANOVA with Dunnett’s multiple comparisons test (*p < 0.05; **p < 0.01; ***p < 0.001). The data represent the mean ± SD.

A growing body of research suggests that astrocyte–neuron and microglial–neuron interactions play important roles in AD progression (Paumier et al., 2022; Kummer et al., 2021). Chronic neuroinflammation and microglial activation can trigger and accelerate pathological alterations in AD (Li et al., 2018). Glial cell activation is a characteristic feature of the AD brain, and GFAP and Iba1 are very well-known markers of glial activation. We examined whether kindled seizures have any effect on GFAP or Iba1 expression in 5×FAD mice. Consistent with previous studies, GFAP and Iba1 immunoreactivity in the 5×FAD group was greater than that in the WT group, and PTZ treatment further increased GFAP and Iba1 protein expression in the 5×FAD group, as shown in Figure 5A–C. Moreover, the WB data revealed that GFAP and Iba1 were upregulated in the hippocampus of 5×FAD mice after PTZ treatment (Figures 5D, E), whereas repeated PTZ simulation did not change GFAP or Iba1 expression in WT mice (Supplementary Figures S1A, B). In addition, the expression of the inflammatory indicator IL-1β also exhibited similar changes, and PTZ insult worsened IL-1β expression in the hippocampus of 5×FAD mice (Figures 5D, E). In conclusion, these data suggest that PTZ kindling exacerbates glial activation in 5×FAD mice.

### PTZ exacerbates AD-like neuropathology in 5×FAD mice through the ERK-DAPK pathway

**FIGURE 6:**
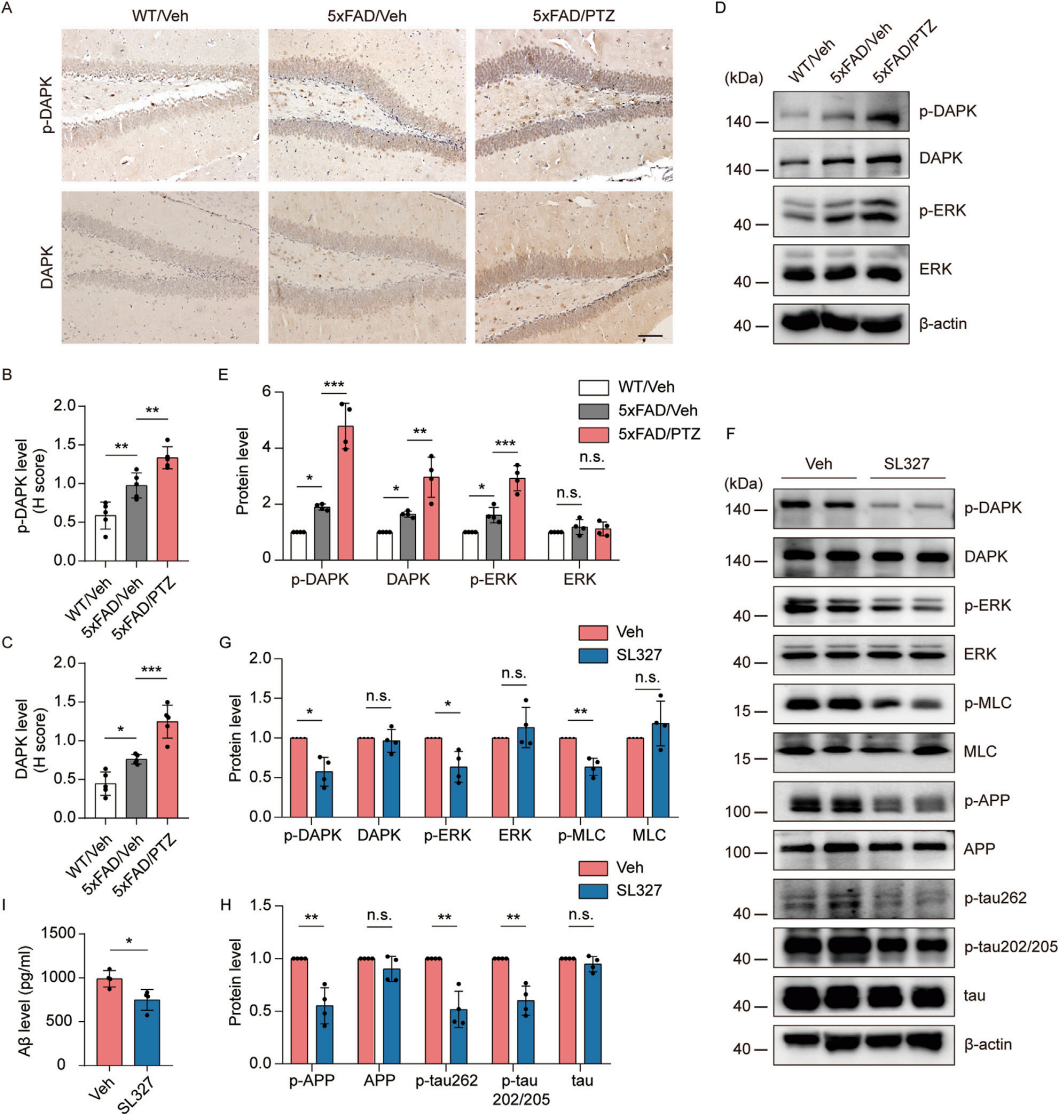
PTZ exacerbates AD-like neuropathology in 5×FAD mice through the ERK-DAPK pathway. **(A–E)** PTZ exacerbates ERK‒DAPK signaling pathway overactivation. **(A)** Immunohistochemical staining with an anti-p-DAPK or anti-DAPK antibodies was conducted on paraffin-embedded hippocampal sections from WT/Veh, 5×FAD/Veh and 5×FAD/PTZ mice. Scale bar, 100 μm. **(B, C)** Quantification of anti-p-DAPK or anti-DAPK staining intensity. N = 5. **(D, E)** Hippocampal lysates from WT/Veh, 5×FAD/Veh and 5×FAD/PTZ mice were subjected to Western blot analysis with anti-p-ERK, anti-ERK, anti-p-DAPK, anti-DAPK or anti-β-actin antibodies. N = 4. Statistical significance was determined by one-way ANOVA with Dunnett’s multiple comparisons test (*p < 0.05; **p < 0.01; ***p < 0.001). **(F-I)** ERK inhibitor SL327 blocked the ERK-DAPK axis and alleviated seizure-induced accelerated neuropathology. 9-month-old PTZ-kindled 5×FAD mice were injected i.p. with 100 mg/mL SL327 or vehicle (60% DMSO/40% saline) and killed 90 min after treatment. **(F–H)** Hippocampal lysates were subjected to Western blot analysis with anti-p-ERK, anti-ERK, anti-p-DAPK, anti-DAPK, anti-p-MLC, anti-MLC, anti-p-APP, anti-APP, anti-p-tau202/205, anti-p-tau262, anti-tau or anti-β-actin antibodies, and **(I)** hippocampal human Aβ42 was detected via ELISA. N = 4. Statistical significance was determined by Student’s t-test (*p < 0.05; **p < 0.01). n.s., not significant. The data represent the mean ± SD.

Since ERK and DAPK activity is induced in both AD and epilepsy, we hypothesized that kindled seizures induced by PTZ exacerbate ERK‒DAPK signaling pathway overactivation in 5×FAD mice. Our experimental data revealed that p-DAPK and DAPK immunoreactivity was elevated in 5×FAD mice compared with WT mice and further increased after PTZ administration (Figures 6A–C). Our WB data revealed that p-ERK and p-DAPK expression was elevated in 5×FAD mice compared with WT mice and further increased after PTZ administration (Figures 6D, E). In addition, DAPK expression was upregulated in the brains of naïve 5×FAD mice compared with those of normal controls, which is consistent with previous reports (You et al., 2017), and DAPK expression was further elevated after PTZ treatment in the 5×FAD group (Figures 6D, E). Since the phosphorylation of DAPK at Ser735 by ERK increases the catalytic activity of DAPK *in vitro* (Chen et al., 2005), we next examined whether the overactivation of DAPK in PTZ-kindled 5×FAD mice was regulated by ERK via the ERK inhibitor SL327. As shown in Figures 6F, G, p-DAPK735 was downregulated, whereas the total DAPK protein level remained unchanged; however, the phosphorylation of the endogenous DAPK substrate MLC decreased after SL327 treatment. Taken together, these data suggest that PTZ kindling causes increased DAPK activity in 5×FAD mice, which is due to an increase in ERK activity. To investigate whether epileptic seizures exacerbate AD-like neuropathology through the ERK-DAPK pathway, we examined two major pathological indicators in kindled AD model mice after SL327 treatment. The WB data revealed that acute SL327 treatment downregulated p-APP and p-tau levels (Figures 6F, H). Moreover, ELISA results revealed that acute SL327 exposure decreased Aβ production (Figure 6I). Taken together, exacerbated Aβ and tau pathology induced by PTZ kindling was regulated by the ERK-DAPK pathway.

### Seizure-induced accelerated AD-like neuropathology and ERK-DAPK overactivation were alleviated by CBZ

**FIGURE 7:**
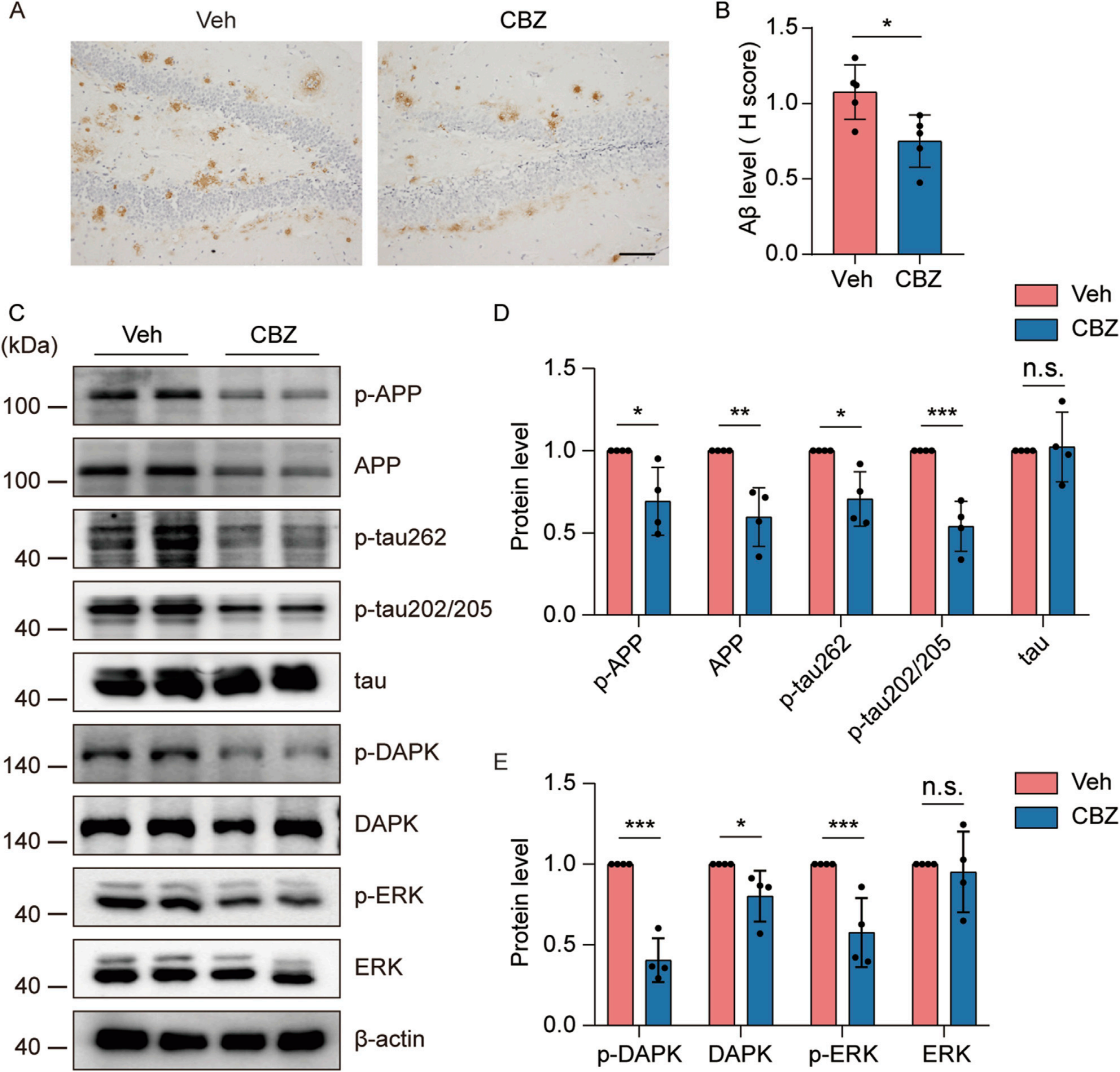
Seizure-induced accelerated AD-like neuropathology and ERK-DAPK overactivation were alleviated by CBZ. 8-month-old PTZ-kindled 5×FAD mice were injected i.p. with 20 mg/kg CBZ or vehicle (10% DMSO) every other day (15 injections for 4 weeks), and brain tissues were harvested for analysis 24 h after the last CBZ treatment. **(A)** Immunohistochemical staining with an anti-Aβ antibody was conducted on paraffin-embedded hippocampal sections from vehicle- or CBZ-treated mice. Scale bar, 100 μm. **(B)** Quantification of Aβ42 staining intensity. N = 5. **(C–E)** Hippocampal lysates were subjected to Western blot analysis with anti-p-ERK, anti-ERK, anti-p-DAPK, anti-DAPK, anti-p-APP, anti-APP, anti-p-tau202/205, anti-p-tau262, anti-tau or anti-β-actin antibodies. N = 4. Statistical significance was determined by Student’s t-test (*p < 0.05; **p < 0.01; ***p < 0.001). n.s., not significant. The data represent the mean ± SD.

CBZ and its derivative (oxcarbazepine) are commonly used antiseizure drugs to reduce spontaneous seizure frequency in the clinic (Craig and Colasanti, 1992). Interestingly, CBZ restored ERK activation in a mouse model of fragile X syndrome (Ding et al., 2020), which is highly susceptible to audiogenic seizures (Sawicka et al., 2016). To investigate whether CBZ can alleviate seizure-induced increases in AD-like neuropathology through the ERK-DAPK pathway, 8-month-old kindled 5×FAD mice were injected i.p. with 20 mg/kg CBZ or vehicle (10% DMSO) every other day (15 injections for 4 weeks) and brain tissues were collected at the indicated time point. Our experimental data revealed that CBZ treatment significantly reduced Aβ plaque (Figures 7A, B), and p-APP, APP and p-tau levels (Figures 7C, D) compared with those in the Veh-treated group. In addition, CBZ decreased p-DAPK, DAPK, and p-ERK levels (Figures 7C, E). Overall, CBZ alleviated seizure-induced accelerated amyloid and tau pathology and ERK-DAPK overactivation in 5×FAD mice.

## Discussion

5×FAD mice are widely recognized models of AD, and PTZ kindling is one of the most commonly used strategies to model TLE and epileptogenesis in animals (Sahin et al., 2022); therefore, 5×FAD mice subjected to PTZ kindling are excellent models for investigating the connection between AD and chronic epilepsy and exploring potential therapeutic targets for these disorders. Our data revealed that 5×FAD mice with chronic epilepsy at the presymptomatic stage exhibited exacerbated AD-like neuropathology and ERK‒DAPK axis overactivation at later stages. Blocking the ERK-DAPK signaling pathway with the ERK inhibitor SL327 or the antiseizure drug CBZ alleviated seizure-induced exacerbation of AD-like neuropathology in 5×FAD mice. Mechanistically, epileptic seizure-induced increases in AD neuropathology are due in part to dysfunction of the ERK‒DAPK signaling pathway.

PTZ-kindled 5×FAD mice presented increased levels of APP, phosphorylated APP and Aβ. Since electrical or chemical stimulation enhances Aβ oligomer formation and accumulation at synapses (Deshpande et al., 2009) and our study revealed that DAPK modulates acute PTZ-induced elevated neuronal excitability (Gan et al., 2021), DAPK may play a role in neural activity-dependent amyloid pathogenesis. APP phosphorylation and metabolism significantly affect Aβ production; in addition, DAPK regulates APP phosphorylation at Thr668 via JNK activation and thus facilitates APP metabolism (Kim et al., 2016). The findings of this study clearly demonstrate that epileptic 5×FAD mice exhibit region-specific increases in DAPK and p-APP668 levels, suggesting that elevated DAPK may promote Aβ generation by increasing APP phosphorylation. BACE1 is a key enzyme in the APP metabolism pathway and has an important role in Aβ production. An interesting finding of the present study is that, consistent with previous research (Yan et al., 2012), epileptic AD mice exhibit considerable BACE1 signaling in the hippocampus, suggesting that chronic epilepsy induces BACE1 upregulation. Since eIF2a and eIF4B phosphorylation positively regulate BACE1 mRNA levels (O'Connor et al., 2008; Bettegazzi et al., 2021) and PERK-mediated phosphorylation of eIF2a is induced in TLE (Gourmaud et al., 2020), epileptic seizures might increase BACE1 mRNA levels via eIF2a phosphorylation. Owing to the presence of multiple phosphorylation sites in the eIF2a protein, it may be regulated by multiple kinases, including DAPK. An important future direction is to investigate whether DAPK can affect the eIF2a-BACE1 axis, thereby affecting APP metabolism to regulate Aβ generation.

p-tau is normally expressed in the developing brain, and tau proteins are found predominantly in axons and support microtubule stabilization, potentially allowing cytoskeletal flexibility and neuroplasticity through phosphorylation (Hernández and Avila, 2007). In AD and other neurodegenerative diseases, tau is hyperphosphorylated and dissociates from microtubules, leading to p-tau aggregation and the formation of tangles in neuronal bodies and dendrites (Chen et al., 2022). This is the first study to show that kindled seizures markedly exacerbated tauopathy in an amyloid mouse model, as epileptic 5×FAD mice presented worsened tau pathology similar to that observed in AD patients with comorbid seizures (Gourmaud et al., 2022). In addition, staining with three antibodies specifically labeling p-tau (Ser202/Thr205, Thr231 and Ser262) revealed changes in kinase-regulated p-tau levels after PTZ kindling. Since DAPK phosphorylates tau at Ser262 (Pei et al., 2015), MAPK phosphorylates tau at Ser202/Thr205 (Drewes et al., 1992), and ERK is a downstream target of MAPK, more studies are needed in the future to clarify the regulatory relationships between the ERK‒DAPK axis and the hyperphosphorylation of tau at different loci in epileptic AD mice. Interestingly, the current experimental studies demonstrate for the first time the regulation of DAPK activity by ERK in an AD model with comorbid seizures. Moreover, we infer that RSK is another upstream target molecule of DAPK in this disease on the basis of the following findings: 1) DAPK has also been proven to be a substrate of RSK (Anjum et al., 2005); 2) the ERK‒RSK axis underlies audiogenic seizure susceptibility in fragile X mice (Sawicka et al., 2016); 3) there is a significant increase in the levels of tau phosphorylated at Thr212 and Ser214 in AD patients with comorbid seizures compared with AD patients without a known history of seizures (Gourmaud et al., 2022); and 4) RSK1 and RSK2 phosphorylate tau at Thr212 and Ser214 (Virdee et al., 2007). Therefore, more experimental studies are needed to elucidate the regulatory network of DAPK in this disease.

In a rodent epilepsy model, PTZ induces synaptic damage and neuronal death, which may involve both excitotoxicity and ischemia-related mechanisms (Gan et al., 2022; Song et al., 2019). In the present study, we report for the first time that synaptic damage in an AD mouse model was worsened by PTZ kindling. PSD95 is a marker of synaptic function, and MDM2-mediated ubiquitination is critical for regulating PSD95 degradation in response to excitotoxins, including NMDA and KA (Colledge et al., 2003; Kim et al., 2020). Given that DAPK phosphorylates MDM2 and affects its protein stability and function, it is necessary to examine whether the DAPK-MDM2 axis regulates PSD95 expression to mediate synaptic damage in response to the excitotoxin PTZ in the future. On the other hand, given that the DAPK-tau interaction mediates synaptic damage (Pei et al., 2015) in stroke and that DAPK-NDRG2 interplay drives neuronal apoptosis in AD (You et al., 2017), DAPK overactivation may also drive these two pathological changes in seizure-induced exacerbation of AD. Finally, identifying new DAPK substrates and interacting proteins involved in this disease is necessary.

In summary, the present study demonstrated that PTZ-induced kindled seizures lead to accelerated Aβ plaque formation, worsened p-tau expression, neuronal and synaptic damage, and exacerbated inflammatory responses and ERK-DAPK overactivation in an AD model mouse. In addition, an acute ERK inhibitor alleviated aggravated amyloid and tau pathology induced by PTZ kindling. Moreover, the antiseizure drug CBZ alleviated seizure-induced accelerated amyloid and tau pathology and ERK-DAPK overactivation in 5×FAD mice. More experiments are needed in the future to verify the role of DAPK in this process, and targeting the ERK-DAPK pathway may hold therapeutic promise in AD patients with comorbid seizures.

## Data Availability

The raw data supporting the conclusions of this article will be made available by the authors, without undue reservation.
