# Supplementary material for: Epileptic seizures induced by pentylenetetrazole kindling accelerate Alzheimer-like neuropathology in 5×FAD mice
Source: Front Pharmacol. 2024 Oct 31;15:1500105. doi: 10.3389/fphar.2024.1500105 (PMC11560768; doi:10.3389/fphar.2024.1500105)

Figure_1E

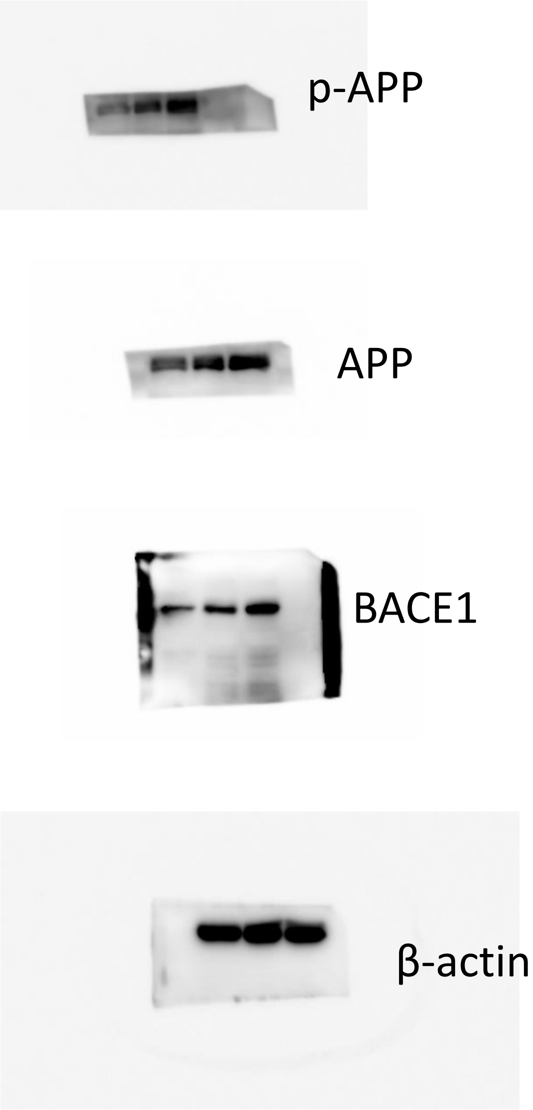

Figure_2E

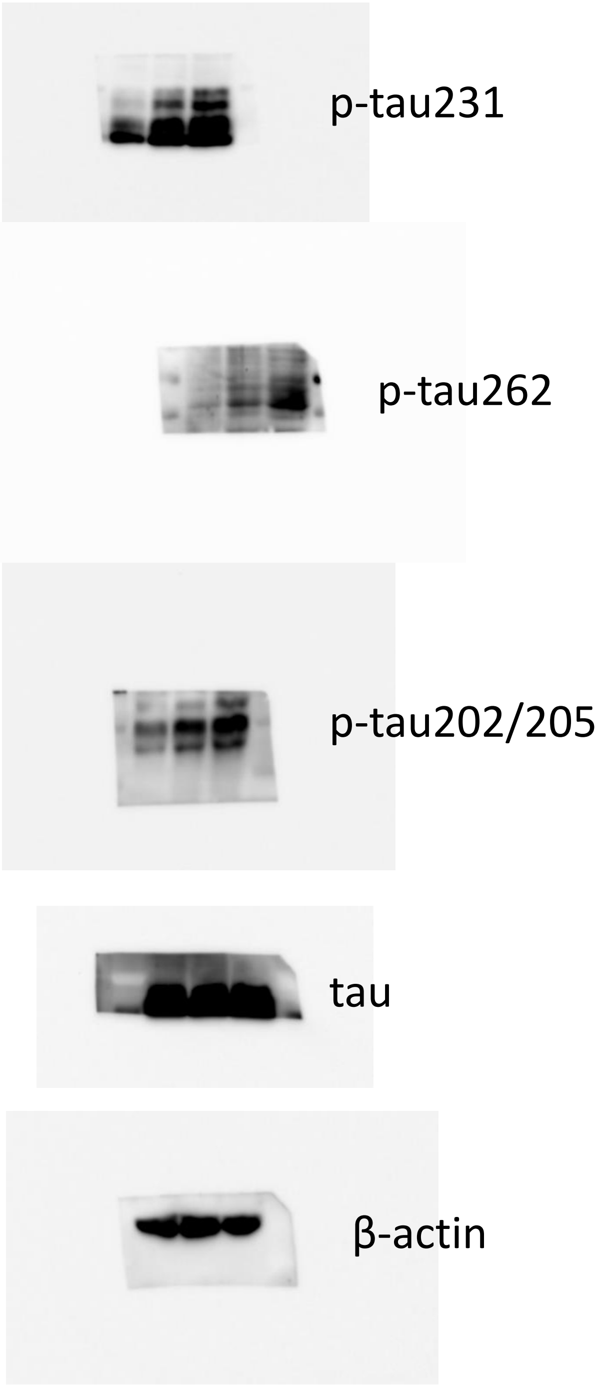

Figure_3D

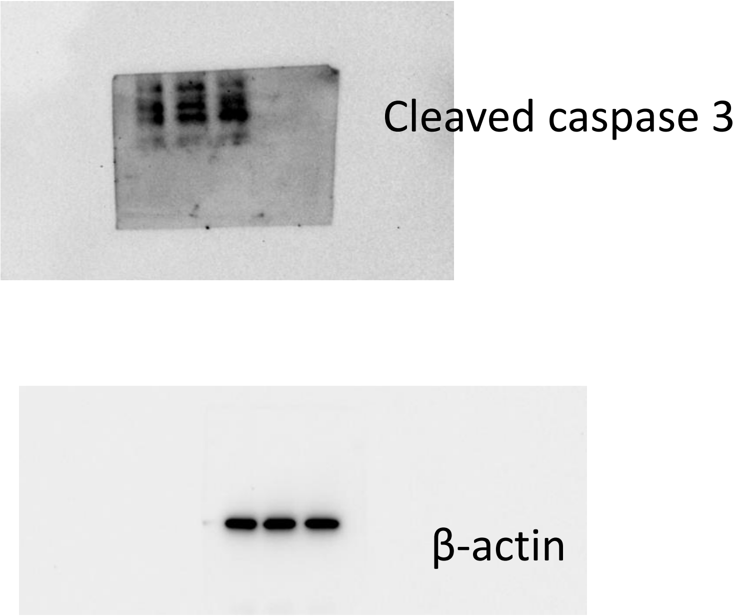

Figure_4D

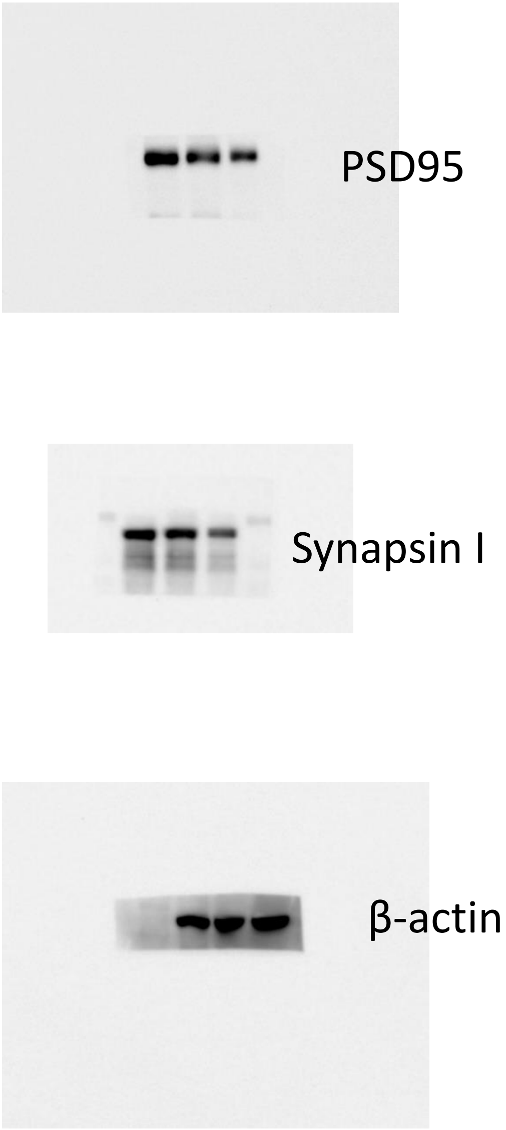

Figure_5D

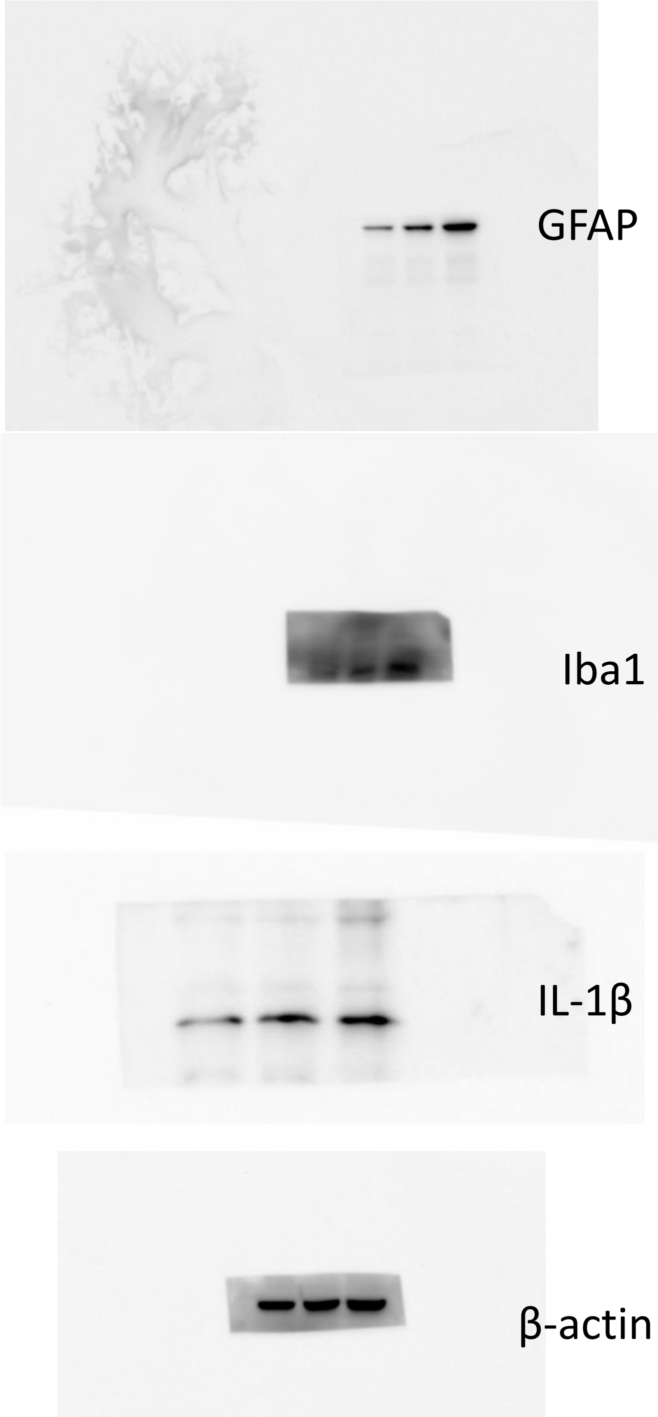

Figure_6D

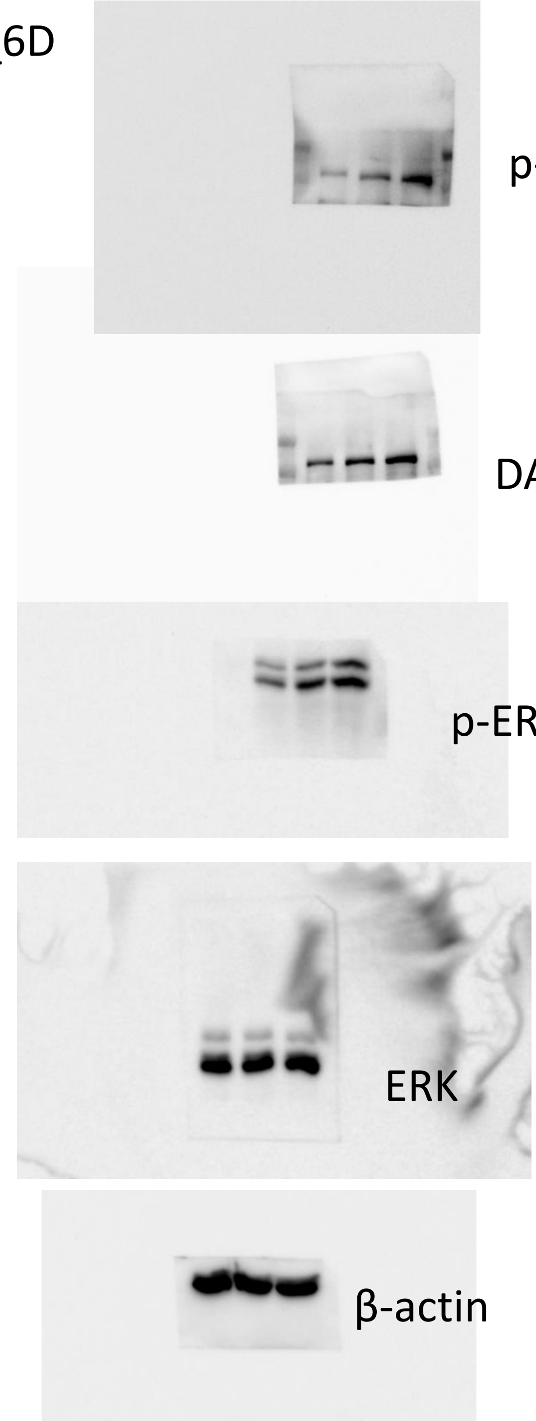

Figure_6F

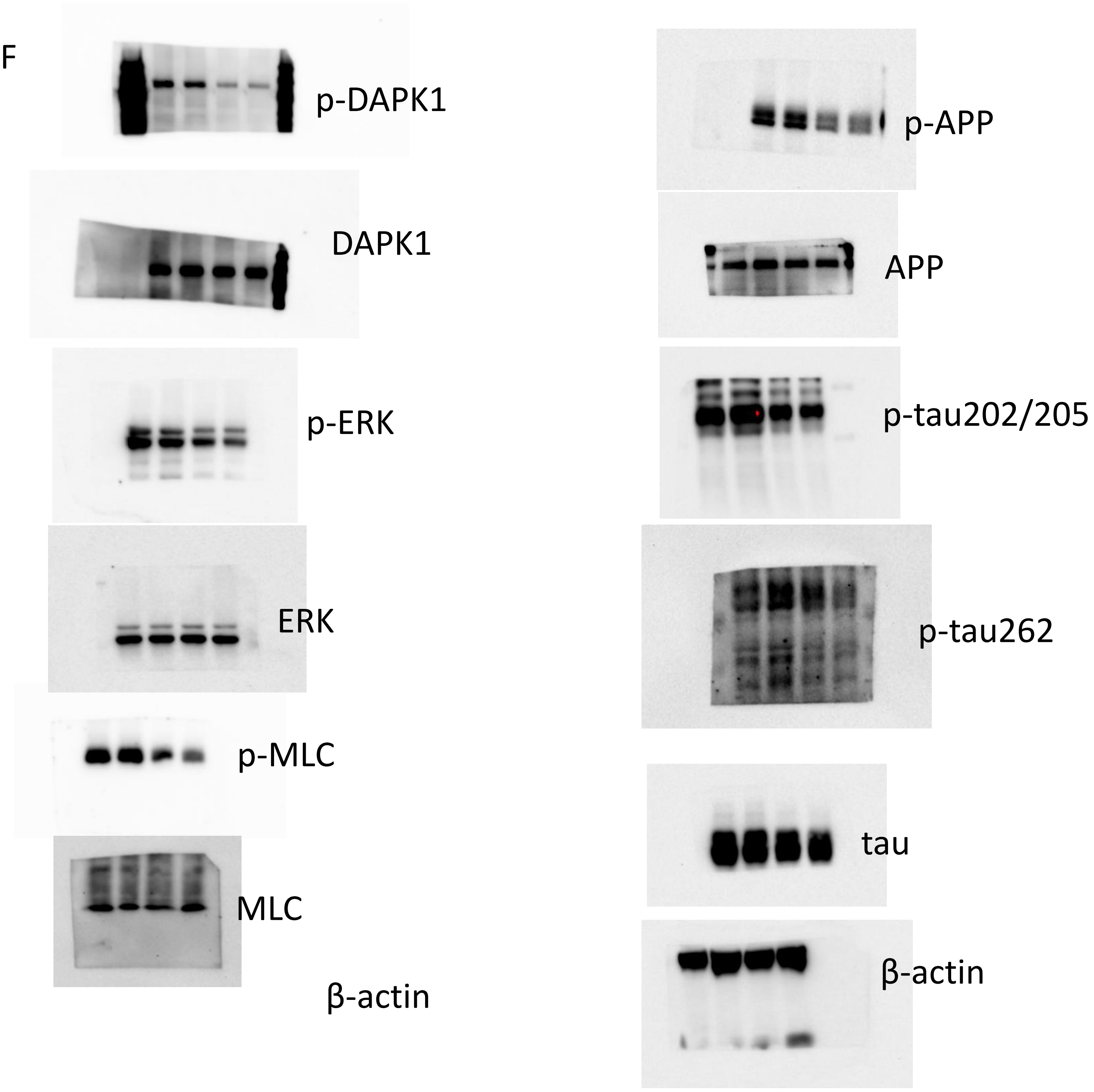
p-DAPK1
DAPK1
p-ERK
ERK
p-MLC
MLC
β-actin
p-APP
APP
p-tau202/205
p-tau262
tau
β-actin

Figure_7C

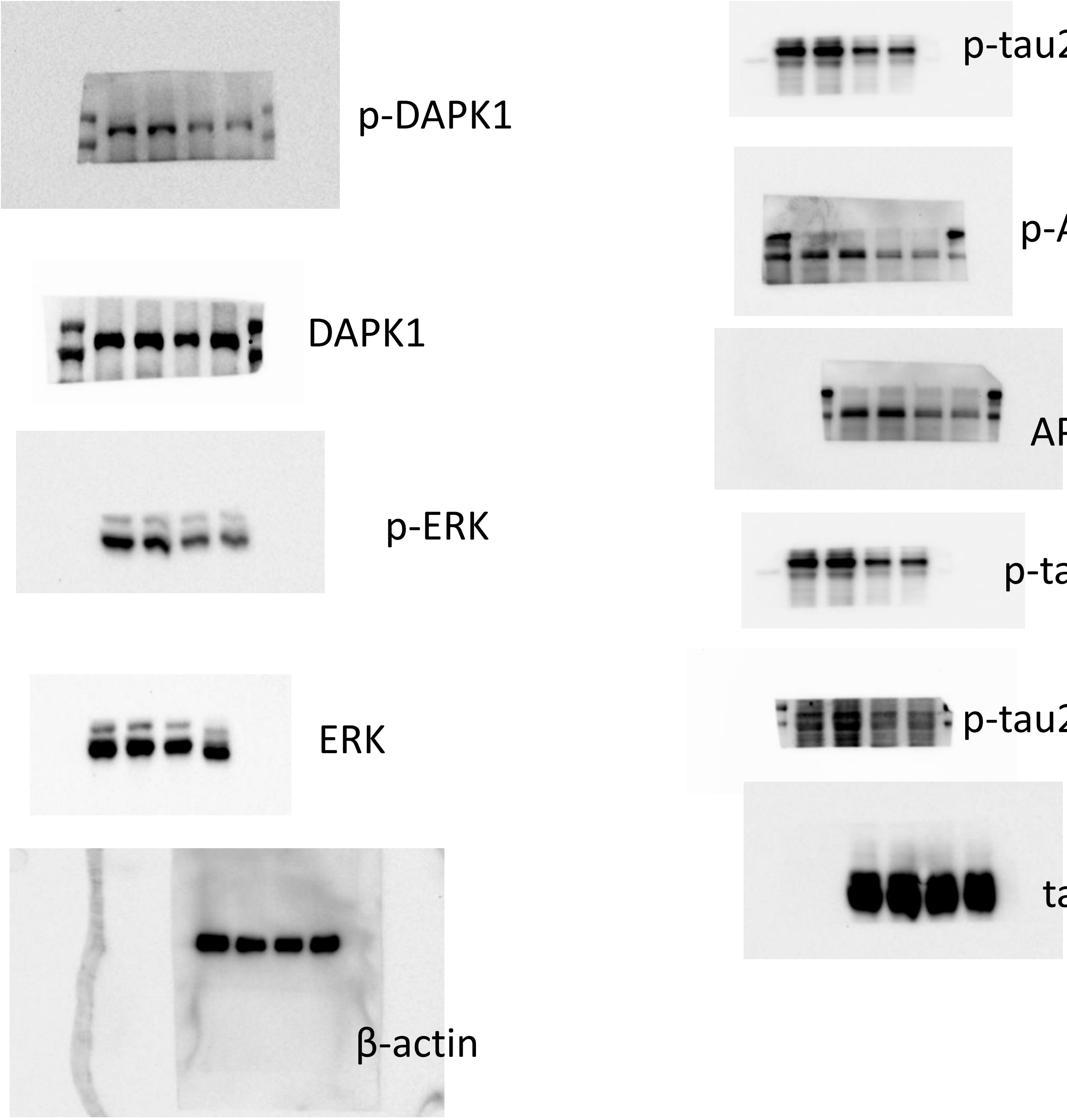
p-DAPK1
DAPK1
p-ERK
ERK
β-actin
p-tau202/205
p-APP
APP
p-tau202/205
p-tau262
tau

Figure_S1A

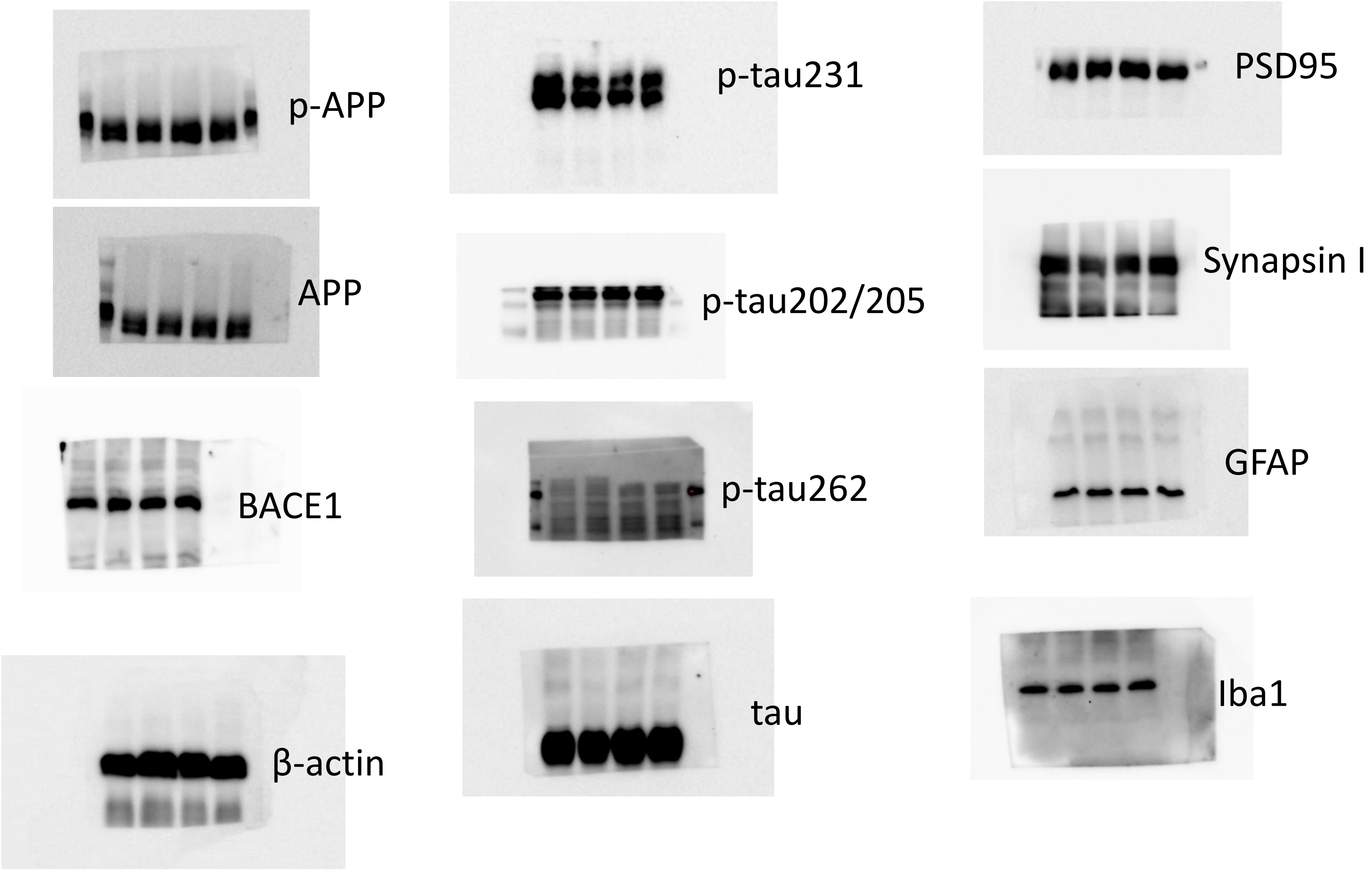
p-APP
APP
BACE1
β-actin
p-tau231
p-tau202/205
p-tau262
tau
PSD95
Synapsin I
GFAP
Iba1

Figure_S2A

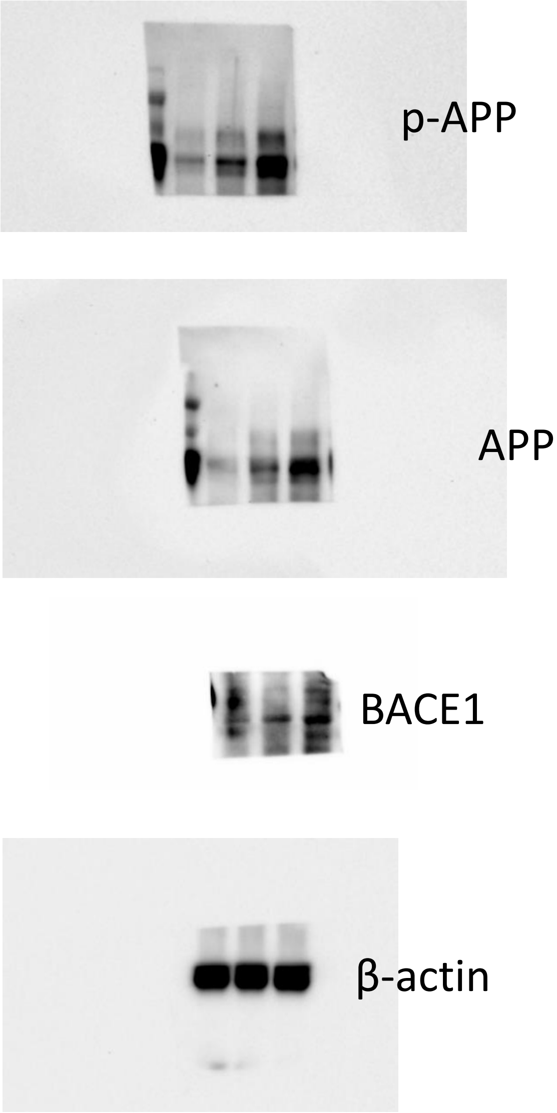

Supplement: Supplementary file 1 [file DataSheet2.PDF]
