# Supplementary material for: Epileptic seizures induced by pentylenetetrazole kindling accelerate Alzheimer-like neuropathology in 5×FAD mice
Source: Front Pharmacol. 2024 Oct 31;15:1500105. doi: 10.3389/fphar.2024.1500105 (PMC11560768; doi:10.3389/fphar.2024.1500105)
Supplement: Supplementary file 2 [file DataSheet1.pdf]

Supplementary Materials

Zou et al.

Epileptic Seizures Induced by Pentylenetetrazole Kindling Accelerate Alzheimer-Like Neuropathology

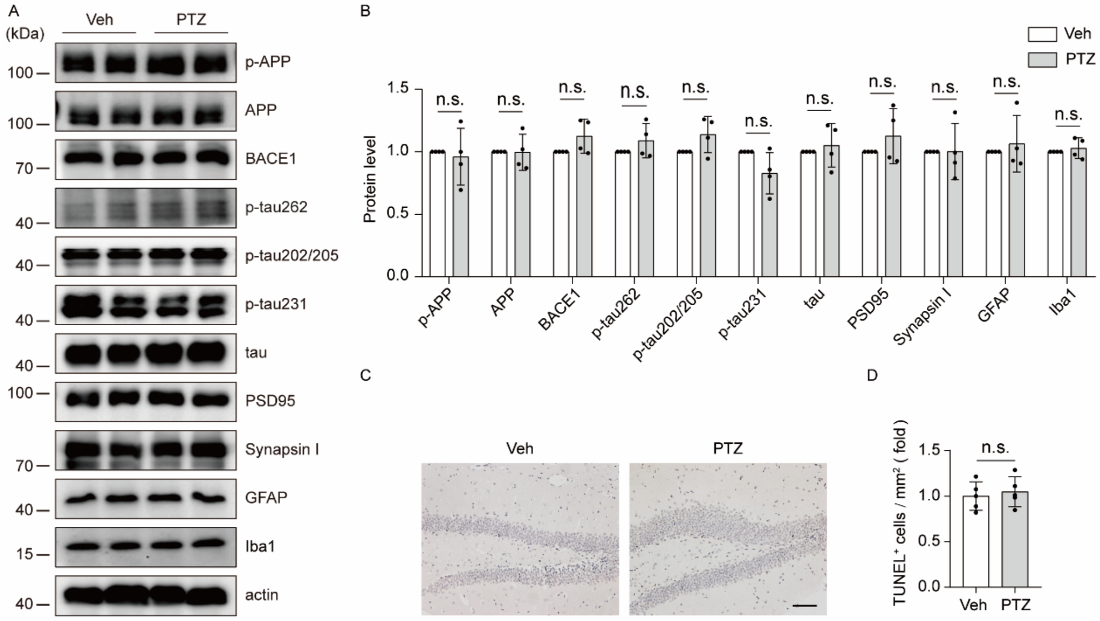

Fig. S1 Repeated PTZ administration cannot induce AD-like neuropathology in WT mice.

WT mice (C57BL/6, P90–P110, n = 6/group) were treated with a subconvulsive dose of PTZ (35 mg/kg) every other day (7 injections for 2 weeks), and brain tissue was harvested for analysis 6 months after the last PTZ treatment. **A-B** Hippocampal lysates were subjected to western blot analysis with anti-APP, anti-p-APP, anti-BACE1, anti-p-tau231, anti-p-tau262, anti-p-tau202/205, anti-tau, anti-PSD95, anti-Synapsin I, anti-GFAP, anti-Iba1 or anti-β-actin antibodies. N = 4. **C** Representative image of TUNEL staining from WT/Veh and WT/PTZ mice. Scale bar, 100 μm. **D** Quantification of the TUNEL staining intensity. N = 5. Statistical significance was determined by Student's t test. n.s., not significant. The data are presented as the means ± SD.

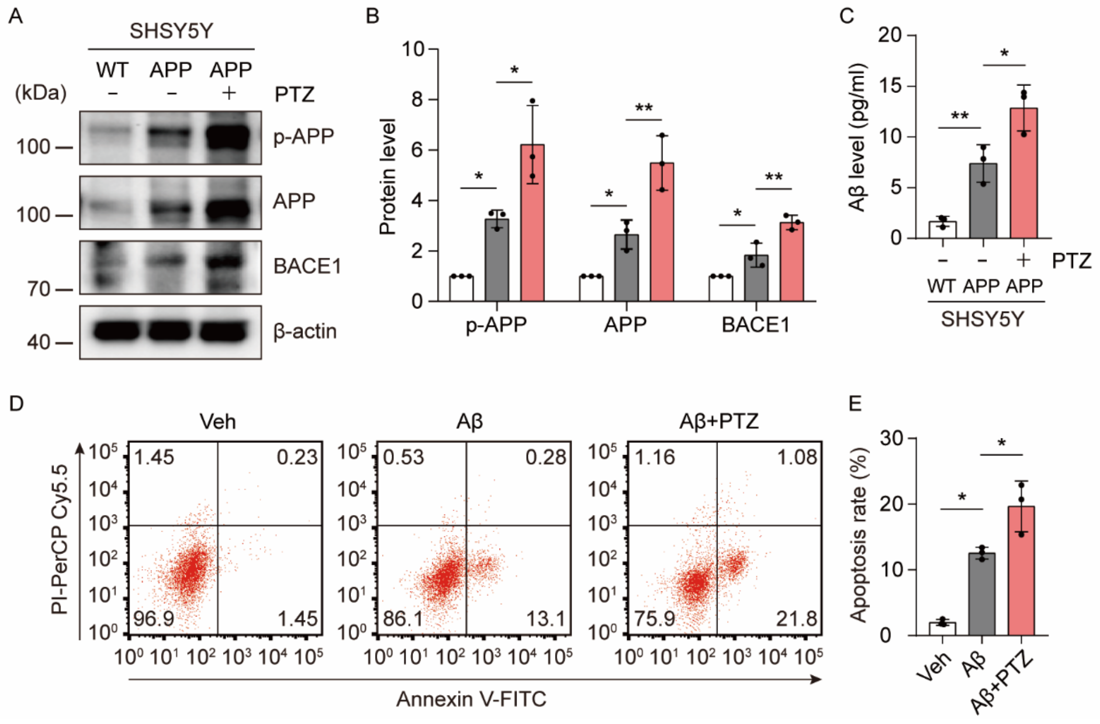

Fig. S2 PTZ administration exacerbated amyloid pathology and neuron death in in vitro AD models

A-C PTZ increased p-APP, APP and BACE1 levels and the generation of Aβ42 in vitro. **(A-B)** SHSY5Y-APP cells were treated with 10 mM PTZ for 24 h. Cell lysates were subjected to western blot analysis with anti-APP, anti-p-APP, anti-BACE1, or anti-β-actin antibodies, the cell culture media were collected, and **(C)** Aβ42 was detected via ELISA. Statistical significance was determined by one-way ANOVA with Dunnett's multiple comparisons test (*$P < 0.05$; **$P < 0.01$). N = 3. **D-E** PTZ increased the degree of cell death induced by Aβ. SH-SY5Y cells were pretreated with 15 μM Aβ or Veh, followed by 10 mM PTZ for 24 h, and cell apoptosis rates were analyzed via flow cytometry. Statistical significance was determined by one-way ANOVA with Dunnett's multiple comparisons test (*$P < 0.05$). N = 3. The data represent the mean ± SD.
